# Tranexamic Acid as a Preventive Strategy Against Periprosthetic Joint Infection in Aseptic Revision Arthroplasty: A Comprehensive Review

**DOI:** 10.7759/cureus.70796

**Published:** 2024-10-03

**Authors:** Sharad Sawant, Sanjay V Deshpande, Bhushan Patil, Hitendra Wamborikar, Vivek H Jadawala, Anmol Suneja, Sachin Goel

**Affiliations:** 1 Orthopaedics, Jawaharlal Nehru Medical College, Datta Meghe Institute of Higher Education and Research, Wardha, IND

**Keywords:** aseptic revision arthroplasty, blood transfusion, hematoma formation, infection prevention, periprosthetic joint infection, tranexamic acid

## Abstract

Periprosthetic joint infection (PJI) is a severe complication following joint replacement surgeries, particularly in aseptic revision arthroplasty, where infection rates are higher compared to primary procedures. The extended surgical time, increased blood loss, and the presence of scar tissue contribute to the higher susceptibility to PJI in revision surgeries. Tranexamic acid (TXA), a synthetic antifibrinolytic agent, is widely used in orthopaedic surgery to reduce intraoperative and postoperative bleeding. By stabilising blood clots and reducing the need for blood transfusions, TXA improves patient outcomes and reduces complications related to excessive bleeding. Emerging evidence suggests that TXA may also play a role in reducing PJI, as minimising bleeding and haematoma formation can reduce bacterial colonisation and blood transfusions are associated with increased infection risks due to immunomodulation. This review explores the potential of TXA as a preventive strategy against PJI in aseptic revision arthroplasty, evaluating its mechanisms, clinical applications, and current evidence. While TXA’s efficacy in reducing blood loss is well-established, its role in infection prevention, particularly through indirect mechanisms such as limiting haematoma formation, warrants further investigation. By incorporating TXA into multimodal strategies aimed at reducing PJI, surgeons can potentially improve patient outcomes and reduce the financial burden on healthcare systems. This review provides a comprehensive examination of the available data on TXA’s role in preventing PJI in revision arthroplasty, with an emphasis on understanding its mechanisms and identifying gaps in current knowledge that require further research.

## Introduction and background

Periprosthetic joint infection (PJI) is a serious complication that can arise following joint replacement surgeries, such as hip or knee arthroplasty. It is characterised by the infiltration of microorganisms, primarily bacteria, into the area surrounding the implanted prosthesis, leading to inflammation, tissue damage, and mechanical failure of the joint [1]. One of the major challenges in managing PJI is the formation of bacterial biofilms on the prosthetic surface, which render the infection resistant to antibiotics and immune system defences. Consequently, PJI is often difficult to treat and may result in chronic infection, extended hospitalisations, and the need for multiple surgical interventions [2]. In the context of aseptic revision arthroplasty, where prosthesis replacement occurs due to mechanical failure or wear rather than infection, the risk of developing PJI is higher compared to primary joint arthroplasty. The incidence of PJI in aseptic revision cases ranges from 1% to 9%, influenced by factors such as surgical complexity, patient comorbidities, and procedure duration [3]. These surgeries typically involve more extensive tissue dissection, greater blood loss, and longer operative times, all of which contribute to a heightened susceptibility to infection. This complication not only worsens patient outcomes but also imposes significant financial burdens on healthcare systems due to the necessity of costly treatments and extended care [4].

Aseptic revision arthroplasty is generally performed when a joint prosthesis fails due to non-infectious causes such as mechanical wear, instability, or periprosthetic fracture. Unlike revisions driven by infection, where sepsis necessitates reoperation, aseptic revisions aim to restore joint function and alleviate pain caused by issues like implant loosening or instability [5]. However, these surgeries are complex and pose unique challenges compared to primary joint replacements. Surgeons must contend with scar tissue, damaged bone, and altered anatomy from the previous surgery, all of which increase the technical difficulty and the likelihood of complications [5]. Compared to primary arthroplasty, aseptic revision surgeries typically involve longer operation times, greater blood loss, and higher complication rates. The need to remove and replace failed components often exacerbates these difficulties [6]. Moreover, revision surgeries carry an increased risk of infection due to prolonged surgical exposure, making PJI a common and serious concern. With the global rise in joint replacements, understanding and mitigating the risks associated with aseptic revision arthroplasty, particularly PJI, is crucial for improving patient outcomes [6].

Tranexamic acid (TXA), a synthetic antifibrinolytic agent, is widely utilised in orthopaedic surgeries to reduce blood loss. Its mechanism of action (MOA) involves inhibiting plasminogen activation, thereby preventing fibrin clot breakdown and promoting stable clot formation [7]. This reduces both intraoperative and postoperative bleeding, making TXA an essential component of blood management strategies in joint replacement surgeries. TXA has been shown to significantly reduce the need for blood transfusions, thereby lowering the risk of transfusion-related complications and shortening hospital stays [7]. Historically, TXA has been used primarily to control blood loss in joint arthroplasty. However, emerging evidence suggests that TXA may also play a role in infection prevention, particularly in the context of PJI [8]. Excessive bleeding during surgery can lead to haematoma formation, which provides an ideal environment for bacterial growth and increases the risk of infection. By reducing bleeding and minimising haematoma formation, TXA may help lower the risk of PJI. Additionally, reducing the need for blood transfusions, which are associated with immune suppression and an increased risk of infection, may offer another benefit in preventing infections [8].

The goal of this review is to provide a comprehensive analysis of TXA as a preventive strategy against PJI in aseptic revision arthroplasty. While TXA’s role in minimising blood loss is well-established, its potential to reduce infection rates, particularly PJI, remains an area of active investigation. This review will explore current evidence supporting TXA’s role in infection prevention, examine the mechanisms by which TXA may reduce PJI risk, and discuss its integration into clinical practice. By evaluating the available data, this review aims to offer insights into how TXA can be utilised not only to decrease surgical bleeding but also as part of a broader strategy to enhance outcomes in aseptic revision arthroplasty.

## Review

Pathophysiology of PJI

PJI is a significant complication that can arise following joint arthroplasty, particularly during revision surgeries. The pathophysiology of PJI involves a complex interplay between bacterial colonisation, biofilm formation, colonisation of patients, and procedure-related risk factors. Understanding these mechanisms is essential for developing effective prevention and treatment strategies [9]. Bacterial colonisation on prosthetics is the initial step in PJI development. Bacteria can adhere to the surface of implants through several mechanisms influenced by the properties of the implant itself. Factors such as the implant's chemical composition and surface roughness play a critical role in bacterial adhesion. For example, hydrophobic surfaces are more likely to promote bacterial colonisation than hydrophilic surfaces. Once attached, bacteria proliferate and form biofilms-structured communities encased in a self-produced extracellular polymeric substance (EPS). These biofilms provide bacteria with a protective environment, rendering them more resilient to host immune responses and antimicrobial treatments [10]. Biofilms confer several advantages to bacteria, notably increased antibiotic resistance and immune responses. The EPS matrix acts as a physical barrier, limiting the penetration of antimicrobial agents and thus diminishing their effectiveness. Furthermore, within biofilms, some bacteria enter a dormant state, forming "persister cells" that are metabolically inactive and capable of surviving high concentrations of antibiotics [11]. Bacteria within biofilms also communicate through quorum sensing, coordinating their behavior to enhance survival against host defenses and therapeutic interventions. Studies indicate that bacteria within biofilms can exhibit antibiotic resistance levels 10 to 1,000 times higher than their planktonic (free-floating) counterparts, complicating treatment strategies as conventional antibiotics are often ineffective against biofilm-associated infections [11]. Several risk factors contribute to the development of PJI, particularly in revision arthroplasty. Host-related factors are significant. For example, older patients often have a diminished immune response, making them more susceptible to infection. Comorbidities such as diabetes mellitus, obesity, and autoimmune diseases further impair wound healing and weaken immune function, increasing infection risk. Patients with compromised immune systems are particularly vulnerable, as their bodies are less capable of fighting off pathogens effectively [12]. Procedure-related factors also impact the likelihood of PJI. Prolonged surgical durations increase the exposure to potential pathogens and may cause greater tissue trauma, elevating infection risk. Excessive blood loss during surgery often necessitates blood transfusions, which are linked to an increased risk of infection due to immune modulation associated with the transfused blood products. Moreover, foreign materials, such as implants, provide an ideal surface for bacterial colonisation and biofilm formation [13]. A history of previous infections is another critical risk factor for PJI. Patients who have previously experienced infections around the joint or with other prosthetic devices may harbor residual bacteria or have altered immune responses that predispose them to new infections. This underscores the importance of thorough preoperative assessments and vigilant management in patients undergoing revision arthroplasty [14].

TXA and its role in surgery

TXA is an antifibrinolytic agent widely utilised in surgical settings for its ability to effectively reduce bleeding. Its pharmacological properties and MOA have made it an essential tool in managing haemostasis across various surgical procedures [15]. TXA functions by inhibiting fibrinolysis, the process by which blood clots are broken down. Specifically, it blocks lysine binding sites on plasminogen, preventing its conversion to plasmin, the enzyme responsible for clot degradation. By stabilising the fibrin meshwork during haemostasis, TXA aids in maintaining blood clots, thereby minimising bleeding during surgery. This mechanism is especially valuable in high-risk surgical environments, where significant blood loss could complicate recovery and heighten the need for transfusions [15]. In orthopaedic surgery, TXA has become standard practice, particularly in total hip and knee arthroplasties. Numerous studies have confirmed its effectiveness in reducing blood loss and the need for transfusions. Whether administered intravenously or orally, TXA has been shown to significantly reduce total blood loss and the postoperative decline in haemoglobin levels [16]. The timing of TXA administration is crucial to its efficacy, with optimal results observed when given early-preferably within three hours of injury or surgical intervention. This proactive approach has led to improved patient outcomes, solidifying TXA as a critical component in modern orthopaedic surgical protocols [16]. While TXA's safety profile is generally favourable, it is important to consider potential risks and contraindications. Though TXA is well-tolerated by most patients, concerns exist regarding its association with thrombotic events, especially in individuals with a history of thromboembolism [17]. However, recent meta-analyses suggest that when used appropriately, TXA does not significantly increase the risk of thrombosis. Key contraindications include patients with a history of thromboembolic disorders or significant renal impairment, as these conditions can interfere with the drug’s clearance and elevate the risk of adverse effects [17]. TXA and its role in surgery are shown in Table 1.

**Table 1:** TXA and its role in surgery TXA: Tranexamic acid; MOA: Mechanism of action

Surgical Setting	MOA	Clinical Benefits	Dosage and Administration	Potential Risks
Orthopedic Surgery [18]	Inhibits fibrinolysis by blocking lysine-binding sites on plasminogen	Reduces blood loss, decreases transfusion requirements, and minimises postoperative haematoma	1g IV pre-incision and 1g IV post-closure or 10-15 mg/kg IV bolus	Thromboembolic events (rare), postoperative swelling
Cardiac Surgery [19]	Reduces bleeding by stabilising clot formation	Decreases transfusions, reduces re-exploration for bleeding	15-30 mg/kg IV bolus followed by infusion or repeated bolus doses	Thrombosis, renal dysfunction (high doses)
Gynecological Surgery [20]	Inhibits enzymatic degradation of fibrin clots	Reduces perioperative bleeding and need for blood transfusion	1g IV pre-surgery or 1-2g IV/PO every 6-8 hours for 1-2 days	Nausea, diarrhoea, dizziness
Spine Surgery [21]	Prevents fibrin degradation, stabilising clots	Reduces intraoperative and postoperative bleeding, transfusion rate	10 mg/kg IV bolus followed by infusion during surgery	Risk of thromboembolic events, seizures (high doses)
Trauma Surgery [22]	Inhibits fibrinolysis, promoting clot stability	Decreases mortality due to haemorrhage in trauma patients	1g IV over 10 minutes, followed by 1g IV over 8 hours	Increased risk of thromboembolic events
Cranial Surgery [23]	Reduces bleeding by stabilising blood clots	Minimises intraoperative blood loss, lowers need for transfusions	10-20 mg/kg IV bolus followed by infusion	Risk of thrombosis, potential for seizures
Urological Surgery [24]	Reduces fibrinolysis, stabilising clots in the urinary tract	Decreases bleeding during and after surgery	1g IV or 25 mg/kg PO pre-surgery, continued postoperatively	Haematuria, renal colic, potential thrombosis
Obstetric Surgery (e.g., C-section) [25]	Inhibits excessive bleeding by stabilising clots	Reduces postpartum haemorrhage and need for transfusion	1g IV at the onset of haemorrhage, repeat if necessary	Nausea, vomiting, risk of thromboembolism
Plastic Surgery [26]	Minimises postoperative bleeding and bruising	Reduces blood loss and need for postoperative drainage	1-1.5g IV preoperatively or 25 mg/kg PO preoperatively	Minor risk of thromboembolic events, nausea
Aseptic Revision Arthroplasty [27]	Reduces bleeding and haematoma formation, potentially reducing infection risk	Decreases blood loss, reduces transfusion requirements, potentially lowers risk of PJI	1g IV pre-incision, with an additional 1g IV post-closure	Minor thromboembolic risk, limited specific evidence

Hypothesis: TXA acid as an adjunct for PJI prevention

TXA has been hypothesised to be critical in preventing PJIs during total joint arthroplasty, primarily by reducing blood loss and modulating immune responses [28]. Haematomas and dead spaces that form during surgery can provide an ideal environment for bacterial colonisation, increasing the risk of infection. Blood accumulation in these spaces facilitates bacterial growth, and by reducing blood loss and limiting haematoma formation, TXA may help mitigate this risk [28]. Excessive blood loss during surgery often necessitates blood transfusions, which are associated with immune modulation that can predispose patients to infections, including PJIs. TXA's efficacy in controlling perioperative bleeding significantly reduces the need for allogeneic blood transfusions. Research suggests that patients who receive TXA are less likely to require transfusions, which correlates with lower postoperative infection risk [29]. By minimising blood loss and haematoma formation, TXA may decrease the likelihood of wound contamination with bacteria. A reduced volume of blood in the surgical site limits the opportunity for bacterial growth, thereby lowering infection risk. TXA's ability to enhance haemostasis not only reduces blood loss but also improves overall surgical outcomes [30]. Stabilising the surgical field can promote better wound healing and reduce complications, including infections. Studies have shown that TXA administration is associated with significantly lower odds of PJIs within 90 days post surgery, supporting the hypothesis that TXA may play a key role in preventing infections through these mechanisms [30]. The potential role of TXA as an adjunct in PJI prevention is detailed in Table 2.

**Table 2:** Hypothesis—TXA as an adjunct for PJI prevention TXA: Tranexamic acid, PJI: Periprosthetic joint infection, RCT: Randomised controlled trial; MOA: Mechanism of action

Hypothesis Component	Description	Rationale	Supporting Evidence	Research Gaps
Primary Hypothesis [27]	TXA administration reduces the incidence of PJI in aseptic revision arthroplasty.	TXA decreases postoperative bleeding and haematoma formation, reducing the medium for bacterial growth and infection.	Observational studies suggest lower PJI rates with reduced haematoma formation.	Limited RCTs specifically evaluating TXA's impact on PJI rates.
MOA [17]	TXA inhibits fibrinolysis, stabilises clots, and reduces postoperative haematoma, which may lower infection risk.	Haematomas provide a nutrient-rich environment for bacteria, and minimising them may decrease the likelihood of PJI.	Studies show that TXA reduces surgical blood loss and haematoma size.	Need for mechanistic studies linking haematoma reduction to infection prevention.
Anti-Inflammatory Properties [31]	TXA may have anti-inflammatory effects that could contribute to a reduced perioperative inflammatory response.	Decreasing inflammation may reduce postoperative complications, including infections.	Some animal and in vitro studies indicate the anti-inflammatory potential of TXA.	Clinical evidence on anti-inflammatory effects in surgical settings is sparse.
Optimal Dosage and Timing [32]	Determining the optimal TXA dose and timing for maximising PJI prevention without increasing thromboembolic risk.	Standardised dosing protocols are crucial for balancing efficacy and safety in different surgical populations.	Protocols for TXA use in primary arthroplasty are established, but less so in revision settings.	There is no consensus on dosing for infection prevention, specifically in revision surgeries.
Combination with Other Strategies [33]	Evaluating TXA in combination with antibiotic prophylaxis, antiseptic measures, and advanced wound care.	Multimodal strategies may provide synergistic effects in preventing PJI.	Combination therapies have shown promise in reducing PJI rates.	Lack of studies evaluating the combined effect of TXA with other preventive measures.
Patient Selection Criteria [34]	Identifying patient populations that may benefit most from TXA use in PJI prevention (e.g., high-risk patients).	Patients with a history of bleeding disorders, high infection risk, or previous PJI may benefit more from TXA.	Some studies suggest varying benefits based on patient demographics and comorbidities.	Need for stratified research to identify high-risk groups for targeted prevention.
Outcome Measures [35]	Measuring PJI rates, wound complications, reoperation rates, and overall recovery in TXA-treated patients.	Comprehensive outcome measures are needed to evaluate the true benefit of TXA beyond just bleeding control.	Some data on reduced complications exist, but specific PJI-focused outcome measures are limited.	Inconsistent outcome reporting in studies limits meta-analysis potential.
Safety Profile [36]	Assessing the risk of thromboembolic events and other complications in patients receiving TXA for PJI prevention.	Balancing the benefits of TXA in reducing PJI with its potential risks is essential for clinical acceptance.	Studies show low thromboembolic risk with standard doses but higher risk in susceptible patients.	Need for long-term safety data, especially in high-risk populations.

Current evidence on TXA in preventing PJI

Current evidence regarding the use of TXA for preventing PJI in arthroplasty is drawn from various clinical trials, retrospective studies, meta-analyses, and systematic reviews. Several investigations have examined TXA’s role in reducing PJI risk. However, much of the focus has been on its impact on blood loss and transfusion rates rather than infection outcomes specifically [37]. For example, a retrospective study noted that administering TXA before skin incision significantly decreased blood loss and the need for transfusions in high-risk patients undergoing cesarean sections. This finding suggests a potential application of TXA in orthopaedic settings, but similar evidence in arthroplasty remains limited [38]. These studies often vary widely in sample size, with some involving large patient populations while others are smaller cohort studies. Common limitations include retrospective study designs, lack of control groups, and inconsistencies in TXA dosing protocols. Importantly, many studies did not list PJI rates as a primary endpoint; instead, they focused on perioperative bleeding and the need for transfusions [38]. Recent meta-analyses have attempted to clarify TXA’s potential in infection prevention by pooling data from multiple studies. While these analyses confirm that TXA reduces blood loss and transfusion rates across different types of joint arthroplasties, they do not establish a direct correlation between TXA use and reduced PJI rates. Though TXA’s efficacy in reducing surgical blood loss is well-supported, its specific impact on infection prevention, when compared to traditional strategies like prophylactic antibiotics, remains inconclusive. Some reviews acknowledge that while TXA improves haemostasis, its role in infection prevention warrants further investigation [39]. Additionally, studies have explored TXA’s use alongside antibiotics and antiseptic measures. The combination of TXA with prophylactic antibiotics might offer enhanced infection control, but definitive conclusions regarding the synergistic effects of this approach have not been established. There are both advantages and disadvantages when considering TXA as a preventive measure against PJIs [33]. On the positive side, TXA is cost-effective, easy to administer (whether intravenously or topically), and significantly reduces blood loss during surgery. However, concerns about the risk of thromboembolic events, especially in patients with pre-existing conditions, remain. Furthermore, the absence of strong evidence directly linking TXA to reduced PJI rates suggests that it should be cautiously integrated into a broader infection prevention strategy that includes antibiotics and other measures [33].

Clinical guidelines and recommendations

Clinical practice guidelines from various orthopaedic societies, including the American Association of Hip and Knee Surgeons (AAHKS), strongly endorse using TXA in total joint arthroplasty to effectively manage blood loss and improve patient outcomes. These guidelines emphasise the importance of TXA for patients undergoing high-risk procedures, particularly revision surgeries, where blood loss is typically more significant compared to primary arthroplasties [40]. While there is broad consensus on TXA's benefits in reducing transfusion rates and associated complications, the guidelines do not explicitly differentiate between its use in aseptic versus septic revision cases. Instead, they focus on TXA’s potential to minimise blood loss and related risks, making it a valuable tool in the surgical management of revision arthroplasties [40]. The integration of TXA into protocols for preventing PJIs during aseptic revision arthroplasty is an evolving area of investigation. Although TXA has demonstrated effectiveness in reducing blood loss and transfusion needs across various types of arthroplasties, its direct impact on PJI rates in aseptic revisions is still under study. Current evidence suggests that minimising blood loss with TXA may indirectly reduce infection rates, as blood transfusions are known to modulate the immune system, potentially increasing susceptibility to infections [27]. Regarding the timing, dosage, and method of TXA administration in revision surgeries, studies indicate that intravenous TXA can effectively reduce total blood loss and shorten hospital stays for patients undergoing both total hip and knee arthroplasties. However, the optimal protocol for TXA in revision surgeries, such as the most effective timing relative to surgical incision and the ideal dosage, remains an area in need of further research [41]. Additionally, the use of TXA in high-risk patients with comorbidities such as a history of venous thromboembolism (VTE), myocardial infarction (MI), or seizures has been carefully scrutinised. Large-scale database studies suggest that TXA does not significantly elevate the risk of VTE in these populations, but it is crucial to assess each patient's risk factors. A multidisciplinary approach is recommended when considering TXA administration, ensuring that patient safety remains a top priority [41]. Clinical guidelines and recommendations for TXA use in surgery are detailed in Table 3.

**Table 3:** Clinical guidelines and recommendations for TXA use in surgery TXA: Tranexamic acid; AAOS: American Academy of Orthopaedic Surgeons; NICE: National Institute for Health and Care Excellence; ESA: European Society of Anaesthesiology; ACOG: American College of Obstetricians and Gynecologists; WHO: World Health Organization; ASA: American Society of Anesthesiologists; RCS: Royal College of Surgeons; ESC: European Society of Cardiology; ANBA: Australian National Blood Authority; SOGC: Society of Obstetricians and Gynaecologists of Canada

Organisation/Guideline	Surgical Context	Recommended Use of TXA	Dosage and Administration	Safety Considerations
AAOS [42]	Total Joint Arthroplasty	Recommended for reducing perioperative blood loss	1-2g IV preoperatively or 10-15 mg/kg IV bolus, followed by infusion	Monitor for thromboembolic complications
NICE [43]	Trauma and Emergency Surgery	Recommended in adults with major trauma and bleeding	1g IV over 10 minutes, followed by 1g IV over 8 hours	Administer within 3 hours of injury for maximum efficacy
ESA [44]	Elective Surgery	Considered for reducing blood loss in major elective surgeries	10-15 mg/kg IV preoperatively or 1g IV before skin incision	Contraindicated in patients with a history of thromboembolic events
ACOG [45]	Obstetric Surgery (e.g., C-section)	Recommended to reduce postpartum haemorrhage	1g IV at the onset of haemorrhage, may repeat if necessary	Low incidence of thromboembolic events; monitor bleeding risk
WHO [46]	Trauma and Postpartum Haemorrhage	Strong recommendation for use in trauma and postpartum haemorrhage	1g IV at onset of bleeding, followed by 1g IV over 8 hours	Best within 3 hours of trauma or haemorrhage onset
ASA [47]	Perioperative Blood Management	Recommended for reducing intraoperative and postoperative bleeding	10-15 mg/kg IV before surgery or 1-2g IV bolus	Avoid in patients with renal impairment or a history of seizures
RCS [48]	Major Surgical Procedures	Considered for reducing blood loss in major surgical procedures	15-30 mg/kg IV or 1g IV bolus, followed by infusion	Careful use in high-risk thromboembolic patients
ESC [49]	Cardiac Surgery	Recommended to reduce perioperative bleeding and transfusions	15-30 mg/kg IV bolus, followed by infusion or repeated bolus doses	Monitor renal function and thromboembolic risk
ANBA [50]	Elective and Emergency Surgery	Recommended as part of multimodal blood management strategies	1-2g IV preoperatively or 10-15 mg/kg IV bolus	Avoid in patients with active intravascular clotting disorders
SOGC [45]	Obstetric Haemorrhage	Recommended for preventing and treating postpartum haemorrhage	1g IV, may repeat once after 30 minutes if bleeding persists	Use with caution in patients with clotting disorders

Future directions and areas for research

The exploration of TXA in the context of PJI prevention during aseptic revision arthroplasty is an emerging and evolving field. Several key areas for future research and ongoing clinical trials are gaining prominence, emphasising the need for a deeper understanding of TXA's role in improving surgical outcomes [8]. A significant gap in the current body of evidence is the absence of robust clinical trials specifically evaluating TXA's efficacy in preventing PJIs during aseptic revision surgeries. Most existing studies focus on TXA’s ability to reduce blood loss and transfusion rates, with little emphasis on its direct impact on infection rates. This gap underscores the necessity for randomised controlled trials (RCTs) that assess TXA’s effectiveness in preventing PJIs, particularly in high-risk patient populations. Future research should address these gaps, offering clearer insights into how TXA can be optimally utilised in clinical practice [51]. Upcoming studies are expected to focus on long-term outcomes associated with TXA use, including the incidence of PJIs over extended follow-up periods. Research targeting specific patient subgroups, such as those with comorbidities or varying American Society of Anesthesiologists (ASA) scores, will provide valuable insights into the optimal use of TXA in different clinical scenarios. Understanding TXA’s effects on diverse patient populations will allow clinicians to tailor treatment approaches, maximising benefits while minimising risks [36]. Beyond orthopaedic surgery, TXA is also being investigated for its potential role in preventing infections in other surgical specialities, including cardiac and abdominal surgeries. The antifibrinolytic properties of TXA may contribute to improved wound healing and reduced infection rates across various surgical settings. This broader application of TXA could lead to enhanced surgical outcomes and a reduction in complications across multiple disciplines [16]. Additionally, research is exploring the potential synergy between TXA and new antimicrobial agents or biofilm-disrupting strategies. Combining TXA with these innovative approaches could improve its effectiveness in preventing infections, particularly in environments where biofilm formation poses a significant challenge. By integrating TXA with emerging technologies, researchers may uncover novel approaches to infection prevention, benefiting patients undergoing a wide range of surgical procedures [52]. Future directions and areas for research on TXA in surgery are outlined in Table 4.

**Table 4:** Future directions and areas for research on TXA in surgery TXA: Tranexamic acid

Research Area	Description	Rationale	Potential Impact	Challenges
Long-term Safety Profile [51]	Investigate the long-term safety of TXA use in diverse patient populations.	Limited data on long-term outcomes, especially regarding thromboembolic events.	Improved understanding of long-term risks and benefits.	Requires large-scale, long-term follow-up studies.
Optimal Dosage and Administration [53]	Determine the optimal dosing and timing for different surgical settings.	Current guidelines vary, and optimal dosing for specific surgeries is unclear.	Standardised dosing protocols could improve efficacy and safety.	Heterogeneity in surgical practices and patient populations.
TXA in High-Risk Populations [54]	Assess the efficacy and safety of TXA in high-risk groups (e.g., patients with bleeding disorders, prior PJI).	High-risk populations may have different risk-benefit profiles.	Tailored guidelines for high-risk patients to maximise safety and efficacy.	Ethical considerations and recruitment challenges.
Mechanistic Studies [55]	Elucidate the exact mechanisms by which TXA may reduce PJI beyond haemostasis.	Current hypotheses on haematoma reduction and anti-inflammatory effects are unproven.	Better mechanistic understanding could refine therapeutic strategies.	Requires complex experimental and clinical designs.
TXA in Combination Therapies [33]	Study the synergistic effects of TXA with antibiotics, antifibrinolytics, or antiseptics.	Combination therapies may provide greater protection against PJI and other complications.	Potentially enhanced efficacy in preventing PJI and other complications.	Interactions between drugs and increased complexity of studies.
Role in Non-Orthopedic Surgeries [56]	Evaluate TXA’s role in preventing surgical site infections (SSIs) in non-orthopaedic settings.	TXA is predominantly studied in orthopaedic surgery; its benefits in other fields are unclear.	The broadened application could lead to improved outcomes across surgical disciplines.	Need for diversified clinical trials.
TXA and Wound Healing [57]	Investigate the impact of TXA on wound healing processes and scar formation.	Limited understanding of how TXA influences wound healing and tissue repair.	Improved postoperative recovery and reduced complications.	Confounding factors and variability in healing responses.
Personalised Medicine Approaches [58]	Explore the use of pharmacogenomics and patient-specific factors to tailor TXA therapy.	Individual responses to TXA vary; personalised approaches could optimise outcomes.	More precise use of TXA, reduced adverse events.	Requires extensive genetic and clinical data collection.
TXA in Outpatient and Ambulatory Settings [59]	Assess the feasibility and safety of TXA use in outpatient surgeries.	Increasing number of surgeries are performed in outpatient settings.	Potential reduction in complications and improved recovery in outpatient surgeries.	Safety and logistical concerns in outpatient settings.
Economic Analysis [60]	Conduct cost-effectiveness studies of TXA use in different surgical contexts.	Understanding economic benefits could support wider adoption of TXA.	Potential for healthcare cost reduction and resource optimisation.	Complex modeling and variation in healthcare systems.

## Conclusions

TXA has long been recognised for its ability to reduce blood loss during orthopedic surgeries, particularly in joint arthroplasty, by stabilising clots and preventing excessive bleeding. Beyond this well-established role, emerging evidence suggests that TXA may offer additional benefits in the prevention of PJI, particularly in aseptic revision arthroplasty. By reducing the formation of haematomas and minimising the need for blood transfusions, both of which are associated with increased infection risk, TXA has the potential to lower the incidence of PJI in these complex surgeries. Although the primary focus of TXA has traditionally been on controlling bleeding, its indirect effects on infection prevention make it a promising adjunct in the comprehensive management of patients undergoing aseptic revision procedures. Further research and clinical trials are needed to strengthen the evidence base and optimise TXA's use as part of a multimodal strategy aimed at reducing PJI, ultimately improving patient outcomes and reducing the healthcare burden associated with infection-related complications in revision arthroplasty.
